# Age-Dependent Detection of Atrial Fibrillation with Implantable Cardiac Monitors in Patients with Cryptogenic Stroke

**DOI:** 10.1055/s-0044-1786015

**Published:** 2024-04-17

**Authors:** Tobias Uhe, Janina Keilitz, Jörg Berrouschot, Rolf Wachter

**Affiliations:** 1 Klinik und Poliklinik für Kardiologie, Universitätsklinikum Leipzig, Leipzig, Germany; 2 Klinik für Neurologie, Klinikum Altenburger Land, Altenburg, Germany

**Keywords:** implantable cardiac monitors, cryptogenic stroke, atrial fibrillation

## Abstract

**Background** Continuous monitoring using implantable cardiac monitors (ICMs) results in atrial fibrillation (AF) detection rates of up to 30% in patients with cryptogenic stroke (CS). Although higher age is an independent risk factor for AF, there are no age-specific recommendations for the implantation of ICM.

**Objective** The aim of this study was to analyze age-related AF rates in patients with CS and continuous rhythm monitoring, to determine the rates of oral anticoagulation (OAC) and recurrent cerebrovascular events (stroke or transient ischemic attack) in patients with ICM-detected AF, and to describe the temporal relationship of AF detection and recurrent cerebrovascular events.

**Methods** In this observational study, patients with CS provided with ICMs were systematically followed. All patients underwent 72-hour electrocardiography monitoring, transcranial Doppler ultrasound, and transthoracic echocardiography prior to ICM insertion. Follow-up included a regular outpatient presentation every 3 months with medical history, physical examination, and interrogation of the ICM.

**Results** One-hundred eighty-six patients (mean age: 65 ± 12 years, 54% female) were included in this analysis. AF was detected in 6, 27, 56, and 65% ( *p* < 0.001) of patients aged less than 60, 60 to 69, 70 to 79, and more than or equal to 80 years, respectively. All patients with AF under 60 years had an impaired left ventricular systolic function. OAC was initiated in 85% of the patients with AF. Recurrent cerebrovascular events occurred in 34 patients of whom 14 had a diagnosis of AF. In nine patients, AF was diagnosed before the occurrence of a recurrent cerebrovascular event.

**Conclusion** AF prevalence increased with age and was absent in CS patients younger than 60 years and with preserved left ventricular ejection fraction. The temporal relationship of AF and recurrent cerebrovascular events was weak.

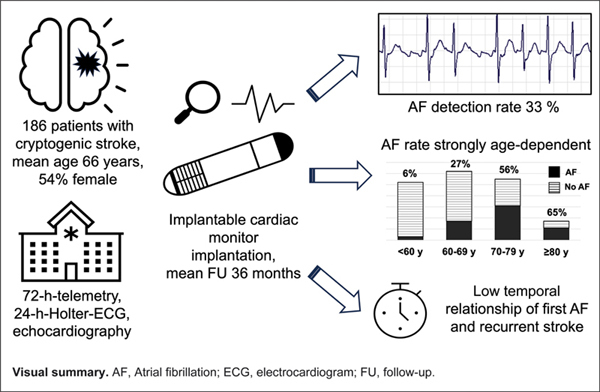

## Introduction

Undetected atrial fibrillation (AF) is considered a relevant cause for cryptogenic stroke (CS). In patients with stroke, prolonged and intensified rhythm monitoring results in higher detection rates of AF. 1 2 3

Continuous rhythm monitoring with the use of implantable cardiac monitors (ICMs) leads to AF detection in up to 30% in patients with CS in the randomized CRYSTAL-AF trial. 2

With odds ratios of 2.1 per decade in men and 2.2 per decade in women, respectively, advanced age is one of the most relevant risk factors for AF. 4 In line with this, AF detection rates using prolonged rhythm monitoring by means of 7-day Holter electrocardiograms (ECGs) in stroke patients increase from 5% in patients younger than 60 years to 39% in patients aged 85 years and older. 5

To date, data regarding AF detection in CS patients in different age groups are scarce and specific recommendations for the implantation of ICMs in patients aged younger than 60 years are lacking.

Moreover, continuous rhythm monitoring has the potential for a detailed investigation of the temporal relationship between the first episode of AF and a recurrent cerebrovascular event. This is of particular interest since a therapeutic change toward oral anticoagulation (OAC) in these patients is usually made after the diagnosis of AF, but the mechanistic link between stroke and AF has been questioned. 6

We, therefore, aimed to analyze the age-related AF rates in patients with CS and continuous rhythm monitoring using ICMs, other relevant risk factors for AF in these patients and the rate of recurrent cerebrovascular events, and the temporal relationship of AF detection and recurrent cerebrovascular events in these patients.

## Methods

From 02/2014 to 11/2021, eligible patients with CS or embolic stroke of undetermined source (ESUS) on a certified German stroke unit were provided with an ICM.

Initially, all patients underwent at least 72-hour bedside-ECG monitoring with a detailed evaluation focused on AF detection by an experienced stroke unit physician. Furthermore, all patients underwent an additional 24-hour-Holter ECG evaluated by an experienced cardiologist and transcranial Doppler ultrasound. In patients aged 60 and younger, thrombophilia, vasculitis, and Fabry disease were excluded by blood tests. All patients underwent transthoracic or transesophageal echocardiography according to current guidelines. A left ventricular ejection fraction (LVEF) lower than 50% was considered as reduced LVEF.

After completion of the baseline examination, the implantation of either the Reveal XT or the Reveal LINQ (Medtronic Inc., Minneapolis, Minnesota, United States) was performed under local anesthesia and the programming was set according to a high sensitivity for AF and as previously described. 7

All patients were instructed to use the Medtronic Home monitor to automatically and manually transmit their ECG data to the CareLink network. An experienced cardiologist analyzed all episodes suspicious of AF.

A structured follow-up was performed by means of a regular outpatient presentation every 3 months and included medical history, physical examination, and a 12-lead ECG. Therefore, stored ECGs were manually analyzed, and AF was defined as any episode of absolute arrhythmia more than 30 seconds according to current guidelines. 8 Both index and recurrent cerebrovascular events (i.e., stroke and transient ischemic attacks) were classified by experienced neurologists and according to current stroke guidelines. 9

This study was in concordance with the Declaration of Helsinki and the local ethics committee (Ethikkommission Klinikum Altenburger Land) approved the study (Ref.: ABG-001/2014). Our manuscript refers to the strengthening the reporting of observational studies in epidemiology (STROBE) guidelines. 10

Continuous variables are given as mean ± standard deviation if normally distributed and as median and interquartile ranges between the 25th and 75th centile if skew distributed. Categorical variables are shown as absolute numbers (%). Continuous data were compared by Student's *t* -test, skew distributed data by Mann–Whitney U test and frequencies by Fisher's exact test or chi-squared test.

AF free survival in different ages and cerebrovascular event recurrence in patients with and without AF were calculated using Kaplan–Meier plots with log-rank test. Data were censored at the time of death or study exit. Thereafter, multivariable analysis using Cox-regression was performed for all parameters associated with AF free survival, e.g., age categories and reduced LVEF, and possible confounders, i.e., parameters with significant differences in baseline characteristics. Skew distributed parameters were log-transformed.

All tests were performed with SPSS Statistics 25.0 (IBM, Chicago, Illinois, United States). *p* -Values less than 0.05 were considered to be significant.

## Results

Between February 2014 and November 2021, 186 patients with CS (41% of them with ESUS) and ICMs were included. The median time from stroke to implantation of ICM was 18 days (11;27). The baseline characteristics are shown in Table 1 .

**Table 1:** Baseline characteristics; *n* = 186; Values are given as mean ± standard deviation, median (interquartile range) or *n* (%)

	Overall ( *n* = 186)	No AF ( *n* = 124)	AF ( *n* = 62)	*p* -Value
Age (years)	65.7 ± 11.5	62.2 ± 11.4	72.8 ± 8.1	<0.001
Female gender	100 (53.8)	72 (58.1)	28 (45.2)	0.096
Comorbidities				
Hypertension	146 (78.5)	90 (72.6)	56 (90.3)	0.005
Heart failure	12 (6.5)	7 (5.6)	5 (8.1)	0.538
Diabetes	57 (30.6)	37 (29.8)	20 (32.3)	0.736
Coronary artery disease	18 (9.7)	12 (9.7)	6 (9.7)	1.000
Previous myocardial infarction	17 (9.1)	11 (8.9)	6 (9.7)	0.857
Dyslipidemia	104 (55.9)	69 (55.6)	35 (56.5)	0.917
Previous stroke or TIA	48 (25.8)	32 (25.8)	16 (25.8)	1.000
CHA _2_ DS _2_ -VASc-score	5 [4;6]	5 [4;6]	6 [5;7]	<0.001
NIHSS at admission	4 [2;6]	3 [2;6]	4 [2;5]	0.807
Stroke localization				0.574
Right	80 (43.0)	51 (41.1)	29 (46.8)	
Left	85 (45.7)	60 (48.4)	25 (40.3)	
Bilateral	21 (11.3)	13 (10.5)	8 (12.9)	
Persistent foramen ovale	19 (10.2)	14 (11.3)	5 (8.1)	0.493
Transthoracic echocardiography				
Left ventricular ejection fraction (%)	63.7 ± 10.9	64.1 ± 9.9	62.9 ± 12.5	0.529
Left atrial diameter (mm)	36.5 ± 6.1	36.3 ± 5.8	36.8 ± 6.7	0.526

Abbreviations: AF, atrial fibrillation; NIHSS, National Institute of Health Stroke Scale; TIA, transient ischemic attack.

**Fig. 1:**
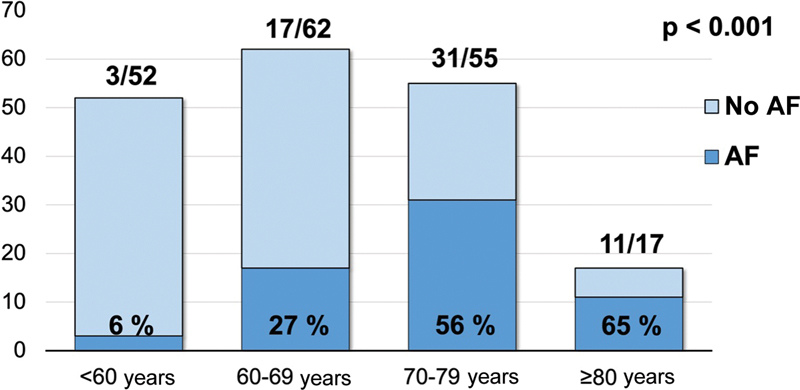
Absolute and relative AF detection in patients aged less than 60 years, 60 to 69 years, 70 to 79 years, and more than or equal to 80 years, respectively. AF, atrial fibrillation; ICM, implantable cardiac monitor.

**Fig. 2:**
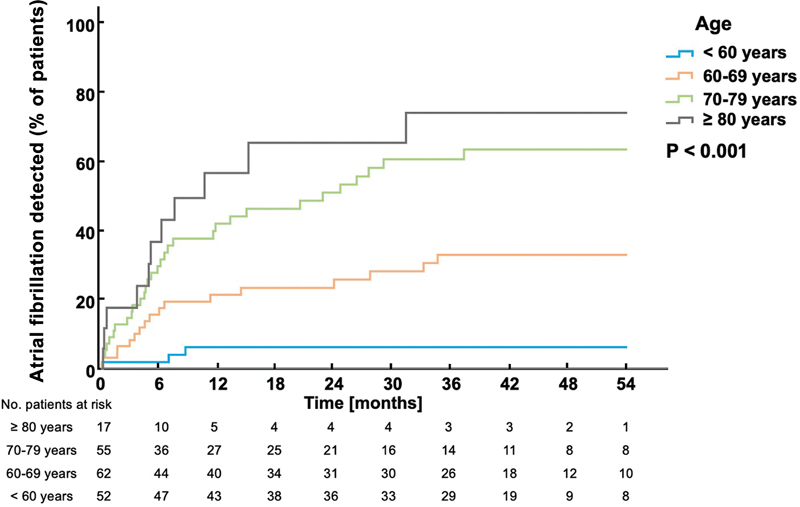
Time to first detection of atrial fibrillation in patients aged less than 60 years, 60 to 69 years, 70 to 79 years, and more than or equal to 80 years, respectively.

The AF detection rate after 12 months of follow-up was 25%. During a mean follow-up of 36 ± 23 months, AF was detected in 62 patients (33%). AF was found in 6 (3/52), 27 (17/62), 56 (31/55), and 65 (11/17; *p* < 0.001) in patients aged less than 60 years, 60 to 69 years, 70 to 79 years, and more than or equal to 80 years, respectively (see Fig. 1 ). Kaplan–Meier curves are shown in Fig. 2 .

All three patients younger than 60 years who had AF during follow-up had a reduced LVEF at baseline. In univariate analysis, significant associations of AF detection were found with age and reduced LVEF less than 50%, while only age remained significant in a Cox regression model including both variables (hazard ratio: 1.079, confidence interval (CI): 1.049–1.110, *p* < 0.001).

After AF detection, OAC was initiated in 53 patients (85%). The median time from AF detection until the start of OAC was 16 days (0;33). Recurrent cerebrovascular events occurred in 34 patients (18%); 14 patients had at least one AF episode and a recurrent cerebrovascular event (41%).

Recurrent cerebrovascular events occurred in 16% of the patients with AF and in 23% of the patients without AF (OR: 1,52; 95% CI: 0.71; 3.26). Ten cerebrovascular events in non-AF patients occurred in the first year, three in the second, third, and fourth; and one in the fifth year, respectively. Of these recurrent events, four were classified as lacunar, three as atherosclerotic, ten as cardioembolic, and 17 as CS (five of them ESUS).

Five patients had more than one recurrent event; these were classified as first cryptogenic and second atherosclerotic in patient 1, first, second, and fourth cryptogenic and third lacunar in patient 2, first cryptogenic and second cardioembolic in patient 3, first and second cardioembolic in patient 4, and first cryptogenic and second due to vasculitis in patient 5.

The suspected etiologies in patients with transient ischemic attacks as the recurrent cerebrovascular event were cardioembolic (3), cryptogenic (2), and lacunar (1).

**Fig. 3:**
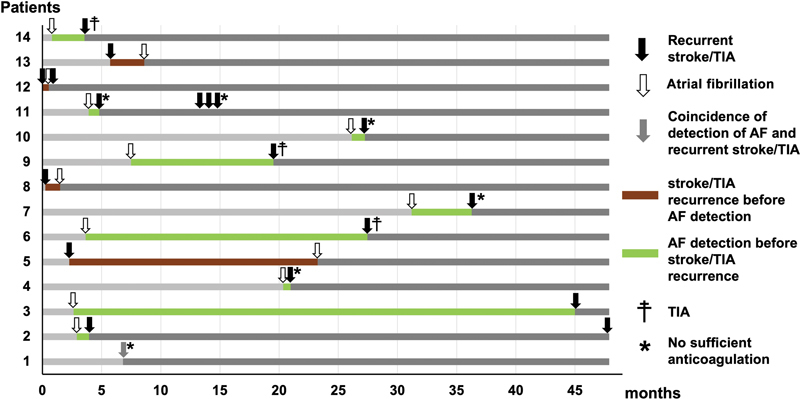
Temporal relationship of first detected atrial fibrillation (AF) and recurrent cerebrovascular events in patients with cryptogenic stroke. Black arrows indicate recurrent cerebrovascular events, white arrows first diagnosed AF, and the gray arrow indicates coincidence of recurrent stroke and first diagnosed AF, respectively. Light gray timelines indicate the time before any of both events, while dark gray lines indicate the time after occurrence of AF and recurrent cerebrovascular event. Timelines are depicted in green in case of AF detection before recurrent cerebrovascular events and in brown in case of recurrent cerebrovascular events before AF detection. Asterisks indicate patients without sufficient anticoagulation after AF detection. Daggers indicate transient ischemic attacks (TIAs)..

Nine of the 14 patients with AF and recurrent cerebrovascular events had an AF episode before the recurrent event and five were anticoagulated at event onset. One patient was admitted to hospital with stroke and an ongoing AF episode. Of note, the recurrent strokes in five patients with AF not anticoagulated occurred relatively early after the first AF detection (after 0, 19, 27, 34 and 155 days, respectively). In four patients, AF was detected after the occurrence of the recurrent stroke. The temporal relationship of the first AF episode and recurrent cerebrovascular events is shown in Fig. 3 .

## Discussion

Our main finding is a strong age-dependent detection rate of AF in patients with CS and continuous rhythm monitoring varying from 6% in patients aged younger than 60 years up to 65% in patients aged 80 and older. Usually, AF detection rates are not reported separately for different age categories but vary according to the mean age of the study population—a finding that is consistent in patients post stroke and patients with cardiovascular risk factors. 2 11 12 13

### AF Detection Rate and Age

After 12 months of follow-up, the overall AF detection rate was 25% that is higher than in previous studies (e.g., 12% in CRYSTAL-AF and 15% in PER-DIEM 2 11 ). In comparison to these trials, patients in our study were older and had more comorbidities. Corroborating our data, both studies reported an increase of AF detection with higher age. A relevant age dependence of AF rates post stroke is not limited to continuous rhythm monitoring by ICMs. Similarly, in intermittent rhythm monitoring using Holter-ECGs, age-dependent AF rates are a common finding as shown with 7-day-Holter-ECGs and AF rates ranging from 5% in patients younger than 65 years to 39% in patients aged 85 and older in the Find-AF study 5 and from 1.4% in patients younger than 65 years to 6.7% in patients aged 65 years and older in the more recent MonDAFIS trial. 14 Our results indicate that continuous monitoring using ICM can be reclined in young patients in the absence of a reduced LVEF less than 50%.

### Oral Anticoagulation for AF

The OAC rate of 85% of the patients with detected AF is slightly lower than the 97% in CRYSTAL-AF and PER-DIEM (100%) after 12 months. This may be attributed to a higher mean age and a higher rate of comorbidities as well as the observational study design because patients with contraindication for anticoagulation were not excluded from our study, but from CRYSTAL-AF and PER-DIEM.

Additionally, the mean follow-up duration of our trial was three times longer than in CRYSTAL-AF and PER-DIEM. OAC rates decrease with longer follow-up duration, because OAC may be discontinued for side effects, for example, bleeding. For instance, OAC rate in CRYSTAL-AF decreased from 97% after 1 year to 90% during follow-up.

### AF and Recurrent Cerebrovascular Events

The overall recurrent event rate of 18% in our study is similar to Kitsiou et al 15 and is usually higher in CS/ESUS patients like ours than non-CS patients and massively elevated compared to patients without stroke (e.g., cardiovascular risk factors only). 16 The absence of a difference in recurrent cerebrovascular event rate in AF versus no-AF patients may be explained by the early initiation of anticoagulation following AF detection. Moreover, our finding of ischemic strokes within 30 days of AF detection predominantly in not adequately anticoagulated patients promotes the immediate initiation of anticoagulation once AF is detected. We found three patients to have their first AF episode in the 30 days before a recurrent stroke (21%), similar to previous studies investigating the temporal relationship between any AF episode and strokes using pacemakers or defibrillators for continuous rhythm monitoring, for example, ASSERT (15%) or TRENDS (28%). 6 17

## Limitations

Our results should be interpreted in the light of some limitations:

First, we performed a single-center study without specific in- and exclusion criteria that pose a risk for a selection bias leading to higher AF detection rates or to the inclusion of healthier patients. Nevertheless, the baseline characteristics and the AF detection rates in our analysis are comparable to similar studies. 15 Second, recurrent cerebrovascular events have not been adjudicated by an end-point committee or an independent neurologist—this could have led to false-positive events. However, in previous stroke studies, an excellent agreement of adjudicators and investigators has been reported and the discrepancies did not affect the study outcome. 18 Third, the duration of AF episodes was not documented in all cases and therefore no analysis of the association of an AF episode duration with recurrent cerebrovascular events was performed. In most cases, the ICM was explanted after the first AF episode and we are unable to give precise information on the progression of AF duration over time.

Finally, our study by design and size is underpowered for any statistical analysis regarding recurrent cerebrovascular event risk or benefit from OAC. The crucial question—if intensified heart rhythm monitoring leads to reduction in recurrent cardioembolism in patients with ischemic strokes—remains unanswered and will be a addressed in the Find-AF 2 study. 19

## Conclusion

The detection of AF by ICMs in CS patients increases with advanced age and is of limited diagnostic value in patients younger than 60 years with preserved ejection fraction. Cerebrovascular events early after AF detection mainly occur in not adequately anticoagulated patients.

## Notes

**What Is Known on This Topic** ?

- Undetected AF is considered a relevant cause for CS.
- Continuous rhythm monitoring using ICM leads to AF detection rates in up to 30% of patients in randomized trials.
- AF is strongly age-dependent; data regarding AF detection in stroke patients younger than 60 years are scarce
- The mechanistic link between first detected AF and stroke has been questioned.
- Continuous rhythm monitoring enables the analysis of the temporal relationship between first detected AF and recurrent cerebrovascular events.

**What Does This Paper Add?**

- The detection rate of AF by ICMs in CS patients increases with advanced age
- In patients younger than 60 years, AF was only detected in patients with impaired systolic left ventricular function.
- Diagnostic benefit of ICM implantation in young patients with CS and preserved LV function is limited.
- Temporal relationship of AF and recurrent cerebrovascular events was weak.
